# Antifungal and antioxidant activity of *Crassocephalum bauchiense* (Hutch.) Milne-Redh ethyl acetate extract and fractions (Asteraceae)

**DOI:** 10.1186/1756-0500-7-244

**Published:** 2014-04-17

**Authors:** Raymond Simplice Mouokeu, Rosalie A Ngono Ngane, Guy SS Njateng, Monique O Kamtchueng, Jules-Roger Kuiate

**Affiliations:** 1 Laboratory of Microbiology and Food Quality Control, Institute of Fisheries and Aquatic Sciences, University of Douala, P.O. Box 7236, Douala, Cameroon; 2 Laboratory of Biochemistry, Faculty of Sciences, P.O. Box 24157, Douala, Cameroon; 3 Laboratory of Microbiology and Antimicrobial Substances, Faculty of Sciences, P.O. Box 67, Dschang, Cameroon

**Keywords:** Antifungal activity, Antioxidant activity, *Crassocephalum bauchiense*, Asteraceae

## Abstract

**Background:**

*Crassocephalum bauchiense* is a flowering plant, found in the West Region of Cameroon. Previous studied has highlighted the antibacterial and the dermal toxicological safety as well as the immunomodulatory activities of the ethyl acetate extract of its dry leaves. As an extension of the previous researches, the current work has been undertaken to evaluate the *in vitro* antifungal and antioxidant activities of *C. bauchiense* dried leaves ethyl acetate extract and fractions.

**Methods:**

The extract was obtained by maceration in ethyl acetate and further fractionated into six fractions labeled F_1_ to F_6_ by flash chromatography. The antifungal activity of the extract and fractions against yeasts and dermatophytes was evaluated using broth microdilution method. Antioxidant activity was evaluated using 2,2-diphenyl-1-picrylhydrazyl (DPPH), nitric oxide (NO) and β-carotene - linoleic acid assays.

**Results:**

The extract (MIC = 0.125 - 4 mg/ml) was found to be more active on dermatophytes and yeasts compared to the fractions. The ethyl acetate extract and fractions exhibited strong scavenging activity on DPPH (CI_50_ = 28.57 - 389.38 μg/ml). The fractions F_3_ and F_6_ expressed best antioxidant activity on DPPH radicals compared to the crude extract.

**Conclusion:**

The results of these findings clearly showed that *C. bauchiense* ethyl acetate extract has a significant antifungal and antioxidant activity. It is therefore a source of active compounds that might be used as antifungal and antioxidant agents.

## Background

*Crassocephalum bauchiense* is a species of flowering plant in the Asteraceae family. Earlier studies on this plant reported the immunomodulatory activity [1] as well as *in vitro* and *in vivo* antibacterial activity [2]. Also the antimicrobial and antioxidant activity of some species of the *Crassocephalum* genus is well established [3-5].

In the last two decades, the incidence of infectious diseases caused by dermatophytes and yeasts has increased considerably worldwide [6]. These diseases are associated to great morbidity among humans with elderly and immuno-compromised patients being the most affected. The situation is more complicated in developing countries with poor hygienic conditions.

Free radical and reactive oxygen species (ROS) are involved in several disorders in human. The harmful action of the free radicals can, however be blocked by antioxidant substances which scavenge the free radicals and detoxify the organism. Current research into free radicals has confirmed that foodstuffs rich in antioxidants play an essential role in the prevention of cardiovascular diseases and cancers [7], and neurodegenerative diseases, including Parkinson’s and Alzheimer’s diseases [8] as well as inflammation. Therefore, plant-derived antioxidants are now receiving a special attention [9].

Plant extracts have been of great interest due to their potential uses as alternative remedies for the treatment of many infectious diseases. Particularly, the antimicrobial activities of plant extracts have formed the basis of many applications [10,11]. The present work has been undertaken to explore the antifungal and antioxidant activities of the *C. bauchiense* leaves extract.

## Method

### Plant material

*Crassocephalum bauchiense* leaves were collected in Dschang (West region of Cameroon). The species was authenticated at the Cameroon National Herbarium in Yaounde where a voucher specimen was deposited with the reference number 7954/SRF/Cam.

### Microorganisms

Fungal species used included eight yeast strains (*Candida albicans* ATCC 1663, ATCC 2091, ATCC 24433, *C. krusei* ATCC 6258*, C. glabrata* CIP 35*, C. parapsilosis* ATCC 22019*, C. tropicalis* ATCC 750 and *Cryptococcus neoformans* IP 95026), four dermatophytes strains (*Trichophyton mentagrophytes* E 1425, *Trichophyton terrestre* E 1501, *Microsporum gypseum* E 1420, *Epidermophyton flocosum* E 1423) and one clinical isolate of *Microsporumaudouinii.* This isolate was a gift from Pasteur Institute, Cameroon.

### Preparation of the plant extract

*C. bauchiense* leaves were dried at laboratory temperature (20 ± 2°C) and ground into fine powder. The dried powdered leaves were macerated in hexane and further in ethyl acetate. For each solvent, the extraction time was 48 hours at ambient conditions. The ethyl acetate extract obtained was filtered and dried at 40°C using a vacuum evaporator. The yield was found to be 10.25 w/w of dry leaves powder. This extract was further fractionated using flash chromatography. In so doing, 50 g of the extract was fixed on silica gel (Merck, 70–230 mesh) and eluted throughout a silica gel column. The elution was done using increasing solvent polarity made of hexane, ethyl acetate and methanol mixture. Fractions of 500 ml for each polarity were collected and evaporated in a vacuum at 40°C, then mixed on the basis of their similarities on thin layer chromatography into six fractions labeled F1 to F6.

### Antifungal activity

Yeast suspensions of about 1.5 × 10^8^ CFU/ml (Mc Farland turbidity standard no. 0.5) were prepared from 48 hours culture in normal saline and diluted in Sabouraud broth culture medium (SDA, Conda, Madrid, Spanish) to obtain a 2.5 10^5^ UFC/ml inoculums [12].

A suspension of conidia was prepared from 10 days old dermatophytes culture on SDA slants (SDA, Conda, Madrid, Espagne) by using sterile physiological saline solution. This solution was added to the slant tube culture and the colony surface was gently swabbed with a sterile bent glass rod to dislodge the conidia from the hyphal mat. The suspension was transferred to a sterile centrifuge tube. Upon centrifugation, the volume was adjusted to 10 ml with sterile normal saline. The final suspension of conidia was counted with a hemocytometer cell counting chamber and adjusted to 10^5^ spores/ml [13].

The *in vitro* antifungal activity of the extract and fractions was performed by determining the minimum inhibitory concentrations (MIC) using broth microdilution method [14]. The stock solutions of *C. bauchiense* extract and fractions were prepared in 5% tween 80. The antifungal susceptibility tests were performed in 96 well microplates. Serial two-fold dilutions of the extract/fractions were performed to obtain a final concentration ranging from 16 to 0.12 mg/ml in a total volume of 100 μl/well. Fungal suspension (100 μl) in Sabouraud broth culture medium was added in all the wells to a final volume of 200 μl/well. The plates were incubated at 35°C for 48 h (yeasts) and at 30°C for five days (dermatophytes). Minimum inhibitory concentrations (MIC) were defined as the lowest concentrations of extract or fraction required to prevent the visual growth of the fungi at the end of the incubation time.

Minimum fungicidal concentrations (MFC) were determined by sub-culturing 10 μl aliquots of the medium drawn from wells which did not show any growth after incubation during MIC assay and incubated further for 48 hours at 35°C (yeasts) or five days at 30°C (dermatophytes) for the appearance of growth. The lowest concentration of the antifungal agent from which negative growth was recorded was considered as MFC [14].

The assays were carried out in triplicate. Nystatin and Griseofulvin were dissolved in 5% acetone and used as positive controls respectively for yeasts and dermatophytes.

### Antioxidant activity

#### *DPPH assay*

The hydrogen atom or electron donation ability of the extract and fractions were measured from the bleaching of the purple colored methanol solution of DPPH. This spectrophotometric assay uses stable radical diphenylpicrylhydrazyl (DPPH) as reagent [15]. One hundred microlitres of various concentrations of the extract and fractions in methanol was added to 900 μl of a 250 mg/ml methanol solution of DPPH. After 30 min incubation period at room temperature, the absorbance was read against a blank at 517 nm. Percent scavenging of the DPPH free radical (I %) was measured using the following equation.

$$I\left(\%\right)=\frac{A_{\text{blank}}-A_{\text{sample}}}{A_{\text{blank}}}\times 100$$

Where A_blank_ is the absorbance of the control reaction (containing all reagents except the test compound), and A_sample_ is the absorbance of the test compound.

#### *Nitric oxide assay*

Nitric oxide, generated from sodium nitroprusside in aqueous solution at physiological pH interacts with oxygen to produce nitrite ions which were measured by Griess reagent. Sodium nitroprusside (10 mM) in standard phosphate buffer saline solution (0.025 M, pH: 7.4) was incubated with different concentrations of extract or fractions (10–1000 μg/ml) and the tubes were incubated at 25°C for 5 hours. After the incubation time, 0.5 ml of incubated solution was removed and diluted with 0.5 ml of Griess reagent and incubated for 10 min. The absorbance was further read at 546 nm. Percentage inhibition (I %) was calculated using the above formula for DPPH assay [16].

#### *β Carotene – linoleic acid assay*

In this assay, antioxidant capacity is determined by measuring the inhibition of the volatile organic compounds and the conjugated diene hydroperoxides arising from linoleic acid oxidation [17]. A stock solution of β-carotene – linoleic acid mixture was prepared as follows: a solution of β carotene was prepared at 15 mg/ml using chloroform (HPLC grade), 75 μl linoleic acid and 600 mg Tween 80 was added to the solution. Chloroform was completely evaporated using a vacuum evaporator. Then, 150 ml distilled water saturated with oxygen (30 min) was added with vigorous shaking to yield an emulsion. A volume of 2680 μl of the emulsion was dispensed into test tubes and 320 ml of the extract or fraction prepared in ethanol at various concentrations was added and the absorbance was obtained at 492 nm immediately and following 105 min incubation at 55°C against blank. The antioxidative capacity (I %) of the extract and fractions was calculated using the following formula:

$$I\left(\%\right)=\frac{{\mathrm{\Delta A}}_{\text{blank}}-{\mathrm{\Delta A}}_{\text{sample}}}{{\mathrm{\Delta A}}_{\text{blank}}}$$

Δ*DO*_*blank *_*is the difference between the absorbance read at 0 and that read at 105 min of incubation of the blank*

Δ*DO*_*sample *_*is the difference between the absorbance read at 0 and that read at 105 min of incubation of the test sample*

In each antioxidant test, the experiment was performed in triplicate. Ascorbic acid and synthetic antioxidant reagent butylated hydroxytoluene (BHT) were used as positive control. The effective concentration of extract and fractions required to scavenge radical by 50% (IC50 value) was obtained by linear regression analysis of dose–response curve plotting between percentage inhibition and concentrations.

#### *Statistical analysis*

Antioxidant experimental results are expressed as means ± SD. The data were analyzed by one way analysis of variance (p < 0.05) and the means separated by Waller Duncan multiple range tests (SPSS program).

## Results

### Antimicrobial activity

The results of the broth microdilution test are presented in Table 1. Minimum inhibitory concentrations of the extract range from 0.125 to 4 mg/ml while those of active fractions range from 0.5 to 8 mg/ml. It was realized that the extract was more active than the fractions. Among the fractions, F3, F4 and F5 were more active. Dermatophytes were more sensitive to *C. bauchiense* ethyl acetate extract with MIC values ranging from 0.125 to 0.250 mg/ml. Among yeasts, *C. albicans* (ATCC 2091) and *C. glabrata* were more sensitive to the extract while *C. parapsilosis* was most resistant. The CMF/CMI ratio was generally less than 4 for both yeasts and dermatophytes.

**Table 1:** **Antifungal activity of *C. bauchiense *ethyl acetate extract and fractions**

	**Parameters**	**Extract (mg/ml)**	**Fractions (mg/ml)**						**Reference (μg/ml)**
			**F**_**1**_	**F**_**2**_	**F**_**3**_	**F**_**4**_	**F**_**5**_	**F**_**6**_	
***Yeasts***									
*C. albicans* 1663	CMI	1	>16	>16	4	2	2	>16	1
	CMF	16	>16	>16	>16	8	16	>16	1
	CMI/CMF	16	-	-	-	4	8	-	1
*C. albicans* 2091	CMI	0.25	>16	>16	4	4	4	>16	1
	CMF	>16	>16	>16	16	16	16	>16	1
	CMI/CMF	-	-	-	4	4	4	-	1
*C. albicans* 24433	CMI	0.5	>16	>16	4	4	4	>16	1
	CMF	2	>16	>16	16	16	16	>16	1
	CMI/CMF	4	-	-	4	4	4	-	1
*C. lusitania*	CMI	2	>16	>16	8	8	4	>16	16
	CMF	>16	>16	>16	>16	>16	16	>16	16
	CMI/CMF	**-**	-	-	-	-	4	-	1
*C. parapsilosis*	CMI	4	>16	>16	4	4	4	>16	16
	CMF	>16	>16	>16	8	16	>16	>16	16
	CMI/CMF	-	-	-	2	4	-	-	1
*C. krusei*	CMI	2	>16	>16	4	4	4	>16	2
	CMF	>16	>16	>16	8	>16	>16	>16	2
	CMI/CMF	-	-	-	2	-	-	-	1
*C. glabrata*	CMI	0.25	>16	>16	4	4	4	>16	16
	CMF	2	>16	>16	8	16	8	>16	16
	CMI/CMF	8	-	-	2	4	2	-	1
*C. tropicalis*	CMI	1	>16	>16	>16	8	8	>16	8
	CMB	>16	>16	>16	>16	>16	>16	>16	8
	CMI/CMF	-	-	-	-	-	-	-	1
*C. neoformans*	CMI	0.5	>16	>16	0.5	0.5	0.5	>16	1
	CMF	>16	>16	>16	16	>16	>16	>16	1
	CMI/CMF	-	-	-	32	-	-	-	1
**Dermatophytes**									
*T. rubrum*	CMI	0.25	>16	>16	0.5	0.25	1	>16	1
	CMF	2	>16	>16	1	0.5	2	>16	16
	CMI/CMF	4	-	-	2	2	2	-	16
*T. mentagrophytes*	CMI	0 .25	>16	>16	0.5	0.5	1	>16	1
	CMF	1	>16	>16	1	1	2	>16	>128
	CMI/CMF	4	-	-	2	2	2	-	-
*M. audouinii*	CMI	0.125	>16	>16	0.5	0.5	0.5	>16	1
	CMF	8	>16	>16	0.5	0.5	1	>16	>128
	CMI/CMF	8	-	-	1	1	2	-	-
*M. gypseum*	CMI	0.125	>16	>16	0.5	0.5	1	>16	8
	CMF	1	>16	>16	1	1	2	>16	128
	CMI/CMF	8	-	-	2	2	2	-	16
*E. flocosum*	CMI	0.125	>16	>16	0.5	0.5	1	>16	1
	CMF	1	>16	>16	0.5	0.5	1	>16	>128
	CMI/CMF	8	-	-	1	1	1	-	-

Yeasts: reference = Nystatine; dermatophytes; reference = Gentamycin; - = undetermined, Fraction F_1_ was found to be inactive.

### Antioxidant activity

*C. bauchiense* ethyl acetate extract and fractions exhibited free radical scavenging activity on DPPH, NO radicals and preserved β – carotene to oxidation (Table 2). The activity was more effective on DPPH radical with the fractions F3 and F6 being the most active. The scavenging activity of the extract on NO radicals and hydroperoxide radicals arising from linoleic acid peroxydation was not quite different. Upon fractionation, the scavenging activity varied considerably within the fractions. With NO assays, the fraction F2 was the most active while in β carotene – linoleic acid assay, the extract was more active than all the fractions. The onset of lipid oxidation in the model system used in this study was initiated by oxygen. The oxygen chelating activities of the extract and fractions corresponded to greater reduction in lipid oxidation.

**Table 2:** **Effect of the *C. bauchiense *leaves ethyl acetate extract and its fractions on *in vitro *free radical scavenging and linoleic acid oxidation**

	**DPPH**	**NO**	**β carotene - linoleic acid**
Extract	55.79 ± 5,40^c^	190.22 ± 1.50^d^	185.62 ± 1.17^b^
F_2_	389.38 ± 3.55^f^	64.67 ± 2.80^a^	897.63 ± 2.00f
F_3_	30.95 ± 2.43^b^	166.25 ± 1.30^c^	306.04 ± 1.50^c^
F_4_	211.94 ± 2.44^e^	90.42 ± 2.00^b^	381.00 ± 1.00^d^
F_5_	104.11 ± 3.20^d^	297.70 ± 1.00^e^	1015.5 ± 2.00^g^
F_6_	28.57 ± 2.11^b^	1102.75 ± 2.10^g^	1086.66 ± 2.50^h^
L ascorbic acid	18.03 ± 4.15^a^	339.70 ± 1.15^f^	849.00 ± 2.13^e^
BHT	20.00 ± 1.00^a^	/	94.00 ± 1.00^a^

Data are expressed as Mean ± S.E.M.; Values for a given column followed by different letter as superscript are significantly different according to Waller Dunkan multiple comparison procedure (at P < 0.05). Fraction F_1_ was found to be inactive.

## Discussion

The *C. bauchiense* ethyl acetate extract was found to express antifungal activity on dermatophytes and yeasts including *T. rubrum*, *T. Mentagrophytes* and *Candida albicans* with MIC varying between 0.125 and 1 mg/ml. This extract was more active than the fractions indicating unnecessary fractionation of the extract for antifungal use. The results are relevant since *Candida albicans* is the leading primary agent causing superficial and often fatal disseminated infections in immunocompromised patients [18]. On the other hand, *Trichophyton rubrum* and *Trichophyton mentagrophytes* are the main cause of athlete’s foot and onychomycoses in human beings, the first one being the most prevalent superficial infection in the developed world population [19] and the second one, an infection that affect 2 – 13% of the population worldwide and up to 30% of groups at high risk such as the elderly people and diabetic patients [20].

The antifungal properties of *C. bauchiense* extract can be linked to the presence of alkaloids, phenols, tannins, triterpenes, and sterols. Indeed, members of these phytochemical classes of compounds are known to possess antimicrobial activities [21]. The fractions F_3_, F_4_ and F_5_ were found to be more active than other fractions. These fractions are similar in that they all contain alkaloids and sterols [2]. These groups of compounds may be responsible for the antifungal activity.

The CMF/CMI ratio was generally less than 4, indicating the fungicidal activity of the *C. bauchiense* extract on both dermatophytes and yeasts [22]. Also, these results may highlight similar mechanism of action of the active compound of the *C. bauchiense* ethyl acetate extract on both yeasts and dermatophytes.

Differences in sensibility among strains could be due to their genetic content. This is an evidence for the necessity of antibiogram prior to antifungal prescription. It is particularly important because inappropriate antifungal drugs enhance fungal resistance [23]. The results thus obtained could explain the traditional use of the *C. bauchiense* leaves in the treatment of gastrointestinal infections.

Free-radicals are generated continuously in the body due to metabolism and diseases [24]. In order to protect themselves against free radicals, organisms are endowed with endogenous (catalase, superoxide dismutase, glutathione peroxidase/reductase) and exogenous (C and E vitamins, carotene, uric acid) defense systems. These defense systems are not sufficient in critical situations such as oxidative stress, contamination, UV exposure, microbial infections, etc. where the production of free radicals significantly increases [25]. There is increasing evidence that indigenous antioxidants may be useful in preventing the deleterious consequences of oxidative stress and there is increasing interest in the protective biochemical functions of natural antioxidants contained in spices, herbs and medicinal plants.

Many available methods are used to evaluate plant extracts for their antioxidant activity. Unfortunately, antioxidant activities of plant extracts cannot be evaluated by any single method, due to the complex nature of phytochemicals [26]. Two or more methods should always be employed in order to evaluate the total antioxidative effects of vegetables [27]. Therefore, three complementary test methods were used to evaluate the antioxidant capacities of the extract and fractions of *C. bauchiense* leaves. Diphenylpicrylhydrazyl (DPPH), Nitric oxide (NO) and β-carotene - linoleic acid. DPPH° radical scavenging method has been proven to be good because its results are not affected by substrate polarity. However, β- carotene/linoleic acid method has the advantage of adding effective *in situ* lipid oxidation to the system. It is a commonly used model to analyze the antioxidant activity of the plant extracts because β-carotene is extremely sensitive to free radical mediated oxidation of linoleic acid [28].

The extract and fractions were able to reduce the stable free radical of DPPH to the yellow coloured diphenylpicrylhydrazine. The potential decrease in the concentration of DPPH radical is due to the scavenging ability of the *C. bauchiense* leaves extract.

In the β – carotene/linoleic acid, oxidation of linoleic acid occurs due to the production of reactive oxygen species formed from halogenated water, this initiates β-carotene oxidation leading to discoloration [29]. The *C. bauchiense* extract and fractions inhibited β- carotene oxidation, suggesting that the extract and fractions inhibit the conjugated diene hydroperoxides arising from linoleic acid oxidation that are known to be carcinogenic. Especially, the extract and non polar fractions exhibited stronger activity than polar ones. This activity could be related to the polarity of tested compound which enable them to be well dissolved in the emulsion.

Nitric oxide (NO) has been associated with a variety of physiological processes in the human body since it was identified as a novel signal molecule [30]. Besides its role in physiologic processes, it also participates in pathogenic pathways underlying a large group of disorders including muscle diseases, inflammatory bowel disease, sepsis and septic shock, primary headaches, HIV-associated dementia, multiple sclerosis and stroke. Additionally, increasing evidence shows that NO modulates neurotoxin induced cell damage and is involved in neuronal cell death in Parkinson’s disease (PD) and other neurodegenerative disorders such as Alzheimer disease [31-33]. This undesired activity of NO is due to its ability to modulate iron catalyzed oxidation reactions such as the O2 – driven Fenton reaction, which produces powerful oxidants such as the hydroxyl radical (·OH) and metalloxo complexes. So, it is very important to investigate the NO scavenging potential of the plant extract. In the present study, the nitrite oxide produced by sodium nitroprusside in standard phosphate saline buffer at 25°C was reduced by the *C. bauchiense* extract and its fractions*.* This extract may have the property to counteract the effect of NO formation and in turn may be of considerable interest in preventing the ill effects of excessive NO generation in the human body. This may be due to the antioxidant principles in the extract which compete with oxygen to react with NO^·^, thereby inhibiting the generation of nitrite. Moreover, the scavenging activity may also help to arrest the chain of reactions initiated by excess generation of NO that are detrimental to human health.

Phenolic compounds are known as powerful chain breaking antioxidants. Phenols are very important plant constituents because of their scavenging ability due to their hydroxyl groups [34]. It has been recognized that flavonoids have antioxidant activity and their effects on human nutrition and health are considerable. The mechanisms of action of flavonoids are through scavenging processes [35]. These substances contain hydroxyl functional groups, responsible for antioxidant effect in plants [35].

The presence of phenols and flavonoids was reported in the ethyl acetate extract of dry leaves of *C.* bauchiense [1]. Thus, the antioxidant potential of the *C. bauchiense* extract and its fractions could be attributed to the presence of these classes of compounds. Indeed, for each antioxidant test, the most active fraction possesses at least one of the two classes of compounds [1].

Fractionation of *C. bauchiense* ethyl acetate extract enhanced antioxidant activity in some fractions. The greatest activity in these fractions could be related to the distribution of phenols and flavonoids compound. Considering the antioxidant activity of the *C. bauchiense* extract on the three systems used and the numerous origins of reactive oxygen species in human body, it may be unnecessary to fractionate this extract for its antioxidant property.

## Conclusion

*C. bauchiense* leaves’ ethyl acetate extract contains compounds with antifungal properties, which can be used as antimicrobial agents in pharmaceuticals and natural therapies of infectious diseases in humans. The antioxidant activity of the *C. bauchiense* extract indicates that it has a protective effect against reactive oxygen species and can therefore be used as a natural preservative ingredient.

## Competing interests

The authors declare that they have no competing interests.

## Authors’ contributions

RSM conceived the manuscript and is the field investigator, RANN design the study and supervised the work. GSSN revised the manuscript. MOK contributed in the field work and also revised the manuscript. JRK supervised the work. All authors read and approved the final manuscript.
